# Molecular Characterization of *Neoseiulus barkeri* Vitellogenin Genes and Vitellogenin Receptor during Reproductive Diapause

**DOI:** 10.3390/insects11040203

**Published:** 2020-03-26

**Authors:** Junqi Jiang, Ying Zhang, Lei Ma, Tingting Niu, Tingting Dong, Ruirui Sheng, Ling Li, Yeyu Xu, Lingyu Xi, Guiting Li

**Affiliations:** Department of Entomology, College of Plant Protection, Anhui Agricultural University, Hefei 230036, China; junqijiang@163.com (J.J.); zhang1007ying@163.com (Y.Z.); mldarker@163.com (L.M.); ntt1993226@163.com (T.N.); ttdong0329@163.com (T.D.); ruiruisheng0307@163.com (R.S.); ll19721056@163.com (L.L.); xyy19721039@163.com (Y.X.); xlyandxzls@163.com (L.X.)

**Keywords:** diapause, expression levels, *Neoseiulus barkeri*, ovary development, vitellogenin, vitellogenin receptor

## Abstract

The relationship between reproductive diapause and the genes related to vitellogenin (Vg) and its receptor (VgR) in insectoid ovarian development is still unclear. Accordingly, in the present study, we used hematoxylin and eosin staining to study the ovarian structure in the predatory mite *Neoseiulus barkeri*, a species that shows promise as a biological pest control agent. Staining revealed the presence of oocytes on ovary surfaces, and the oocytes were deposited as yolk granules through the intake of Vg and other nutrients with the development of the ovary. Development of the ovary stopped at the oocyte stage in diapausing adult mites, and this stage presented the same characteristics as the first day of adulthood in non-diapause female adults, where oocytes with nutrient cells, but no yolk granules are observed. In order to further explore the effects of the Vg gene and its receptor on reproduction, the sequences of the *N. barkeri* vitellogenin genes NbVg1, NbVg2, NbVg3, and NbVgR were analyzed using bioinformatics, and the expression levels of the NbVgs and the VgR at different developmental stages were determined by quantitative polymerase chain reaction (qPCR). The results showed that the NbVgs and NbVgR have complete domains and that the positions of many conservative regions and conservative motif are consistent. The expression levels of the NbVgs and NbVgR were highest in the ovipositional period, followed by those in the preovipositional period. The expression levels of the NbVgs and the VgR in non-diapause female adult mites were significantly higher than those in reproductive diapause female adult mites.

## 1. Introduction

*Neoseiulus barkeri* (Acari: Phytoseiidae) is a polyphagous Phytoseiid mite that preys on a wide range of targets, such as thrips, whiteflies, spider mites, and eriophyoid mites [1,2,3]. It is widely distributed in the United States, Europe, Israel, and Japan, while in China, it is mainly distributed in Jiangxi, Hunan, Guangdong, and Yunnan. It has a short life span, it is easily cultivated, and it is prolific. Accordingly, it is considered to be a highly promising biological control agent [4]. For instance, it has been used in the biological control of *Frankliniella occidentalis* on agricultural crops [5]. However, high winter mortality negatively affects the ability of Phytoseiidae mites to control target species [6].

In colder climates, *N. barkeri* overwinters by means of female reproductive diapause. Moreover, Phytoseiid mites must mate in order to lay eggs. *N. barkeri* show mating behavior in the deutonymph period. Our earlier studies revealed that the mites enter the reproductive diapause stage when the temperature falls to 18 °C and an 8L/16D light-cycle occurs. However, the involvement of the yolk-precursor protein vitellogenin (Vg) and its receptor (VgR) in ovarian development and reproductive diapause in *N. barkeri* is unclear.

There are currently limited data on ovarian development during reproductive diapause in Phytoseius mites, which are a subfamily of mites in the Phytoseiidae family. Several studies have reported that the main characteristic of reproductive diapause in Phytoseius mites is that they do not lay eggs, or, more accurately, that mites in reproductive diapause have longer preoviposition periods [7]. However, very few physiological and molecular studies on the cessation of reproductive activity in diapause Acarine species have been reported [8]. As limited examples, Swirski and Wysoki studied the ovarian development of Phytoseius mites under low temperature and short-light cycles. In that study, female adult mites raised in a short-daylight cycle (L/D = 8:16) had undeveloped eggs in their ovaries, much like female mites found in natural conditions in December and January, when they are dormant. However, the female mites raised in a long-daylight cycle (L/D = 16:8) had ovaries that developed normally and produced hatchable eggs, much like female mites found in natural conditions in March. In addition to reproductive diapause, overwintering female adults were found to have flattened dorsal abdomens during the ovipositional period. Conversely, the abdomens of fertile females were round and enlarged, and round eggs were observed. In another study, significant differences coloration between the two types of female mites was observed [9]. Moreover, female adult *Thuja occidentalis* in diapause are milky white with a granular and uniform texture throughout their body cavities, whereas the non-diapause female adults are pink to light red in color, have slightly reddened backs, and have developed eggs that can be observed in the ventral area of the terminal body [10].

In many vertebrates and invertebrates, Vg plays an important role in promoting growth and development [11]. Vg is mainly synthesized in the fat body and then secreted into the hemolymph, finally entering developing oocytes through endocytosis mediated by their VgRs. Receptor-mediated endocytosis is essential for normal eukaryotic cellular functions, including the uptake of nutrients (such as low-density lipoproteins or transferrin) and the recycling of membrane proteins [12,13]. Vg is stored as vitellin and releases synthesized amino acids during embryogenesis, providing material and energy for the development of embryos and ovaries [14].

Recent studies have shown that Vg is polygenic and that some species express one or more Vg genes. For example, *Nilaparvata lugens* has only one Vg gene [15]; *Gallus gallus* has three Vg genes [16]; *Xenopus laevis* has four [17]; *Tetranychus urticae* has four [18]; and *Caenorhabditis elegans* has six Vg genes [19]. It has also been demonstrated that Vg is evolutionarily conserved with similar domains and sequences, such as the lipoprotein N-terminal domain (LPD_N), unknown functional domain (DUF1943), Von Willebrand factor D domain (VWD), cleavage site (R/KXXR), and C-terminal GL/ICG motif [20].

VgR is a member of the low-density lipoprotein receptor (LDLR) superfamily and a key receptor for Vg uptake. It also plays an important role in oocyte maturation [21]. Lipoproteins are transported into their target cells by VgR, providing a variety of nutrients to support oocyte development [22]. Members of the LDLR superfamily have many common structural elements, including (i) epidermal growth factor-like repeats (EGF-R); (ii) ligand binding domains that consist of cysteine repeats (LBDs); (iii) the transmembrane domain (TMD); (iv) the internalization signal; and (v) the O-glycan domain in the C-terminal cytoplasmic tail. Studies have shown that VgRs are not only found in invertebrates such as insects, mites, ticks, shrimps, crabs, and nematodes, but also in vertebrates, such as fish, frogs, and chickens [23].

## 2. Materials and Methods

### 2.1. Source of Tested Insects

*Tyrophagus putrescentiae* were used as an alternative prey for the large-scale breeding of *N. barkeri*. They were provided by the Key Laboratory of Biological Control, Ministry of Agriculture, Institute of Plant Protection, Chinese Academy of Agricultural Sciences. They were fed wheat bran in an artificial climate box (MGC-300H, Blue pard, China) at 25 ± 1 °C, relative humidity (RH) 80% ± 5%, and L/D = 14:10 h. 

*N. barkeri* were provided by the Key Laboratory of Biological Control, Ministry of Agriculture, Institute of Plant Protection, Chinese Academy of Agricultural Sciences and fed *T. putrescentiae* as raised above. The conditions in the artificial climate chambers were 25 ± 1 °C, RH 80% ± 5%, and L/D = 14:10 h for non-diapause *N. barkeri*, and 18 ± 1 °C, RH 80% ± 5%, and L/D = 8:16 h for diapause *N. barkeri*.

### 2.2. Collection of Insects

A collection device was constructed using a 40 W incandescent lamp, a wire, a funnel (7 cm in diameter), gauze, and a test tube rack, as shown in Figure S1. A water isolation platform was constructed from a culture dish (9 cm in diameter), a filter paper, a black plastic film, and a sponge. A mixture containing wheat bran, *T. putrescentiae*, and *N. barkeri* was spread on the funnel, and a layer of gauze was laid at the upper opening of the funnel. An incandescent lamp was pointed at the top of the funnel and the water isolation platform was placed at the bottom. The mites move towards the water isolation platform. In this manner, the light, heat, and water tropisms of the mites were exploited to select mites in different developmental states. An anatomical mirror was then used to select the target mites, which were isolated by careful use of a writing brush or soft brush. The selected mites were placed on a new water isolation platform for future use.

### 2.3. Developmental Period of *N. barkeri* under Diapause and Non-Diapause Conditions

A transparent plexiglass block (30 mm × 20 mm × 2 mm) with a circular hole (r = 5 mm) in the middle was sealed with parafilm on one side (30 mm × 20 mm) and covered with a plexiglass block (30 mm × 20 mm × 1.5 mm) on the other side. A foldback clip was used to fix the two glass blocks to prevent the mites from escaping. A mite was placed in each feeding room, and the food was changed every day to ensure its survival.

### 2.4. Abdominal Morphology of Female Adult Mites

Diapause and non-diapause female adult *N. barkeri* in different ovary-development states were selected and gently swept into a 1.5 mL centrifuge tube with a writing brush, soaked in 1 mL lactic acid solution (Sinopharm, Shanghai, China), and then left at room temperature. The next day, an appropriate *N. barkeri* was selected and placed on a concave glass slide. Then, 100 µL of 50% lactic acid solution was dripped into it. This was heated with an alcohol lamp to make the lactic acid solution boil slightly, and then placed at room temperature. Under an anatomical mirror (ES-18BZL, Motic, China), the mite was flattened, covered lightly with a glass slide, and its abdominal morphology was observed under a trinocular stereo microscope (AE2000Met, Motic, China).

### 2.5. Ovarian Section

Female adult mites at different stages were fixed at room temperature for 24 h, and then washed and dehydrated with increasing concentrations of alcohol. The tissues were embedded in paraffin wax blocks. The wax blocks were cut into 4 micron thick slices by a slicing machine (EM UC7, Leica, Germany) and stained with hematoxylin and eosin (H&E). After dewatering and sealing, they were observed under a transmission electron microscope (HT7700, Hitachi, Japan). Casevier 2.0 software (3DHISTECH, Budapest, Hungary) was used for processing the images.

### 2.6. RNA Extraction and cDNA Synthesis

Total RNA of *N. barkeri* from different-aged mites was extracted using Trizol reagent (Invitrogen, CA, USA) following the manufacturer’s recommended procedure, and stored at −80 °C for subsequent tests. Then, 1 and 3 μL aliquots of the isolated RNAs were separately analyzed on 1.2% agarose gels by staining with ethidium bromide to determine the integrity of the RNA bands. The OD280 and OD260 values for the RNA samples were obtained using a Nanodrop 2000 Ultramicro spectrophotometer (Thermo Fisher, New York, USA). The typical A260/A280 absorbance ratios of the total RNA fell in the range of 1.8–2.0. A PrimeScript RT reagent kit with a gDNA Eraser (Takara, Dalian, China) was used to isolate the total RNA for the first chain of the cDNA.

### 2.7. Bioinformatics Analyses 

The gene sequences used in the bioinformatics analysis were obtained from the National Center of Biotechnology information (NCBI) (https://www.ncbi.nlm.nih.gov/). SMART (http://smart.embl-heidelberg.de/) was used to identify structural domains, and SIGNALP 3.0 (http://www.dbs.dtu.dk/service/SIGNALP) was used to predict signal peptides. The COMPUTE PI/MW program (http://web.expasy.org/compute_pi/) was used to predict the molecular weight and isoelectric points (pI) of the derived protein sequences. ClustalX2 was used to edit amino acid sequences, and a phylogenetic tree was constructed using MEGA 7.0 software (Tempe, AZ, USA) by the neighbor-joining method with 1000 bootstrap replications.

### 2.8. Quantitative Real-Time Polymerase Chain Reaction (PCR)

Using the Trizol reagent method (Takara, Dalian, China), RNA was extracted from female *N. barkeri* of different ages. Quantitative real-time reverse transcription polymerase chain reaction (qRT-PCR) analysis was performed on a BIO-RAD CFX96 touch q-PCR system (BIO-RAD, Hercules, California, USA) using SYBR^®^ Premix ExTaq^TM^ II (Tli RNaseH Plus) (Takara). The 20 μL reaction volume contained 10 μL SYBR Premix Ex Taq II, 0.5 μM of each primer, 20 ng cDNA template, and nuclease-free water. The thermal conditions were as follows: one cycle of 95 °C for 30 s, 40 cycles of 95 °C for 5 s, and 55 °C for 20 s. Three replicates were performed for each sample, and the relative levels of gene expression among the different samples were measured by the 2^−ΔΔCt^ method. Expression of the actin gene of *N. barkeri* was used as an endogenous control for the normalization of the expression data [24]. The primers used in this section are listed in Table 1.

### 2.9. Statistical Analysis

The relative levels of gene expression among the *N. barkeri* diapause and non-diapause strains were calculated by the 2^−△△ct^ method, and three replicates were performed for each sample. Data Processing System (DPS) software version 7.5 was used to analyze the qPCR results for the NbVgs and NbVgR at different developmental stages [25]. Differences among multiple samples were compared by one-way analysis of variance (ANOVA) with the Duncan new multiple range method, and the level of significance was set at *p* < 0.05. All figures were prepared using GraphPad Prism 5 (GraphPad Software Inc., San Diego, CA, USA).

## 3. Results

### 3.1. Developmental Periods of *N. barkeri* under Diapause and Non-Diapause Conditions

**Figure 1:**
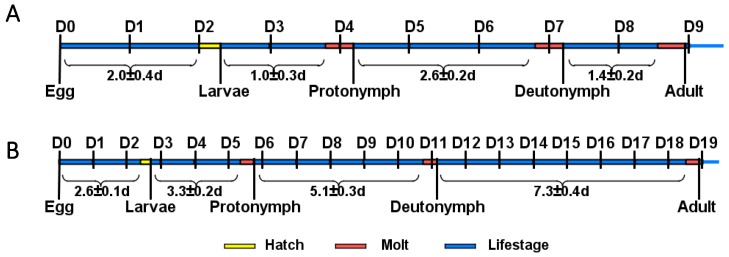
*N. barkeri* development. (**A**) Developmental period of *N. barkeri* under non-diapause conditions. (**B**) Developmental period of *N. barkeri* under diapause conditions. D = days, long blue bars denote life-stage, short yellow bars denote hatching, and short red bars denote molting.

Under non-diapause conditions, *N. barkeri* undergo three molts, the time intervals of which are as shown in Figure 1A, and the total immature period is 8.42 d. *N. barkeri* can develop into adults at 18 °C. The development period of the diapause mite is slightly prolonged, and the total immature period is 18.57 d (Figure 1B). The molting time is delayed with decreasing temperature. The number of moltings does not change over the whole development period.

### 3.2. Analysis of Ovarian Development

**Figure 2:**
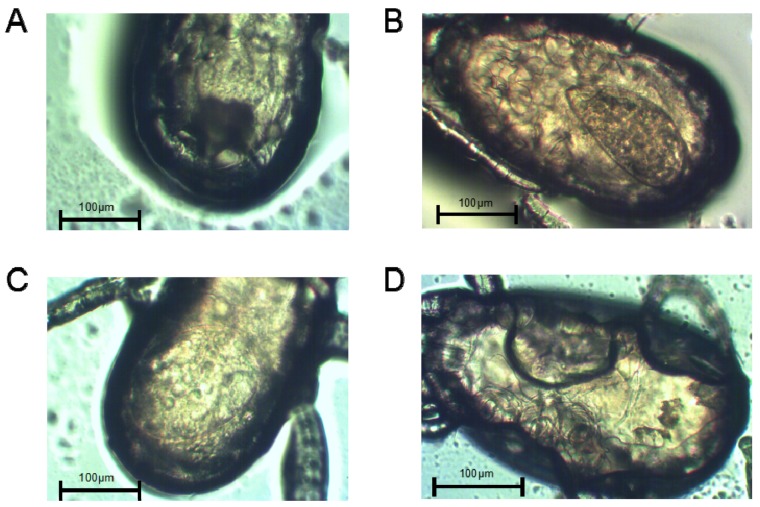
Abdomen morphologies and the ovaries of non-diapause *N. barkeri* adults reared at 25 °C (**A**–**C**) and those of diapause adults reared at 18 °C (**D**). (**A**) Day 1 of the non-diapause female adult mite stage. (**B**) Day 2. (**C**) Day 3. (**D**) Day 5 (diapause stage). Scale bar = 100 μm.

The abdominal morphologies of lactic-acid-treated non-diapause female adult and diapause female *N. barkeri* were observed. On the first day of the female adult stage, the ovaries of the non-diapause female mites have just begun to develop (Figure 2A). On the second day, the ovaries of non-diapause female ovaries show obvious enlargement (Figure 2B). On the third day of the female adult mite stage, the ovaries of the non-diapause female mites are well developed, egg outlines are visible, and yolk particles are deposited (Figure 2C). On the fifth day of the diapause female adult mite stage, there are no mature ovaries, and no yolk particles are deposited (Figure 2D).

**Figure 3:**
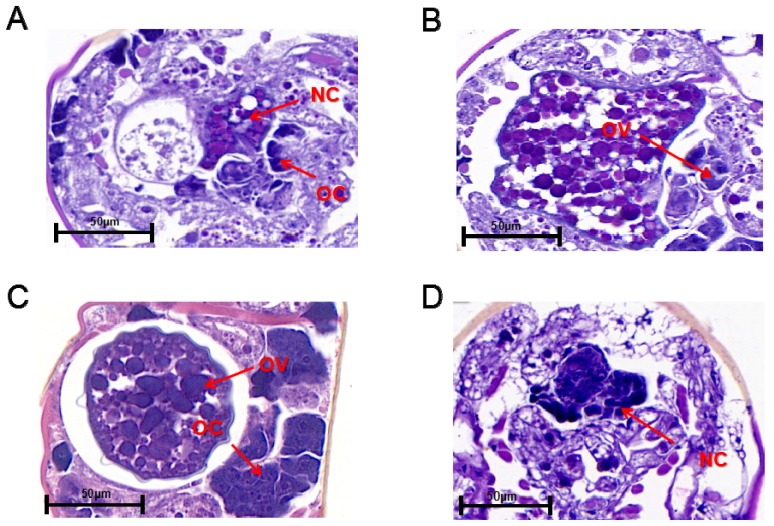
Longitudinal sections of the ovaries of non-diapause *N. barkeri* adults reared at 25 °C (**A**–**C**) and those of diapause adults reared at 18 °C (**D**). (**A**) Day 1 of non-diapause female adult mite stage. (**B**) Day 2. (**C**) Day 3. (**D**) Day 5 (diapause stage). OC, oocyte; NC, nurse cell; OV, ovum. Scale bar = 50 μm.

The ovaries of non-diapause and diapause female adult *N*. *barkeri* (mated) were observed. On the first day of the non-diapause female adult mite stage, oocytes with one nucleus and nurse cells with two nuclei are observed, but no yolk granules are observed (Figure 3A). On the second day of the non-diapause female adult mite stage, the yolk cells develop into yolk granules in the ovary. At this stage, the eggs of the female adult mites are incompletely developed and appear wrinkled (Figure 3B). On the third day of the non-diapause female adult mite stage, the ovaries of the non-diapause female mites are filled with yolk granules and have distinct dividing lines. The eggs are surrounded by eggshell and separated from the ovaries (Figure 3C). On the fifth day of the female adult mite stage, the ovaries of the diapause female mites shrink and there are two nurse cell nuclei in the ovaries without yolk granules (Figure 3D).

**Figure 4:**
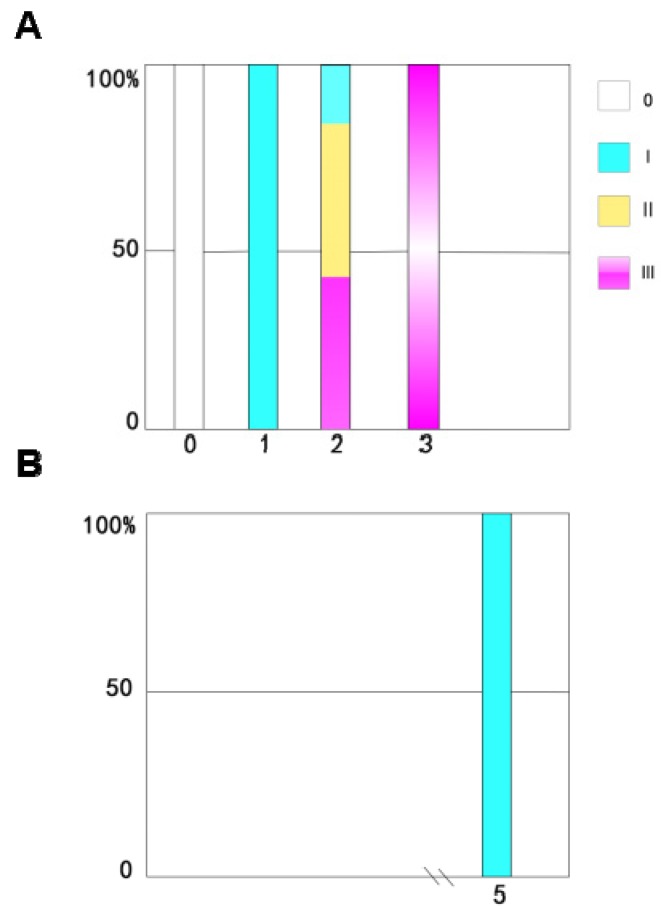
Time course of ovarian development in the non-diapause adults of *N. barkeri* reared at 25 °C (**A**) and the stages in ovarian development in the diapause adults reared at 18 °C (**B**). The sample size for each histogram is 30.

The time course of ovarian development in the non-diapause female adult mites shows that the nymph is equivalent to the 0 stage after maturation (i.e., it has not yet developed into a female adult mite). Ovarian development is in phase I on the first day for adult-female-stage mites, the ovaries have just begun to develop, and oocytes and nutrient cells have appeared. On the second day, ovaries have begun to develop in more than half of the female mites, and most are in stage Ⅱ. By the third day, all the female adult mites have laid eggs. The ovaries have matured and the ovaries are in stage Ⅲ (Figure 4A).

Monitoring the time course of ovarian development in the diapause female adult mites revealed that all the female adult mites have immature ovaries. The ovarian development of diapause female adult mites stops at stage I, and they present ovarian characteristics that correspond to those in non-diapause female adult mites on the first day of the adult stage (Figure 4B).

### 3.3. Bioinformatics Analysis

#### 3.3.1. Bioinformatic Analysis of Vgs

**Figure 5:**
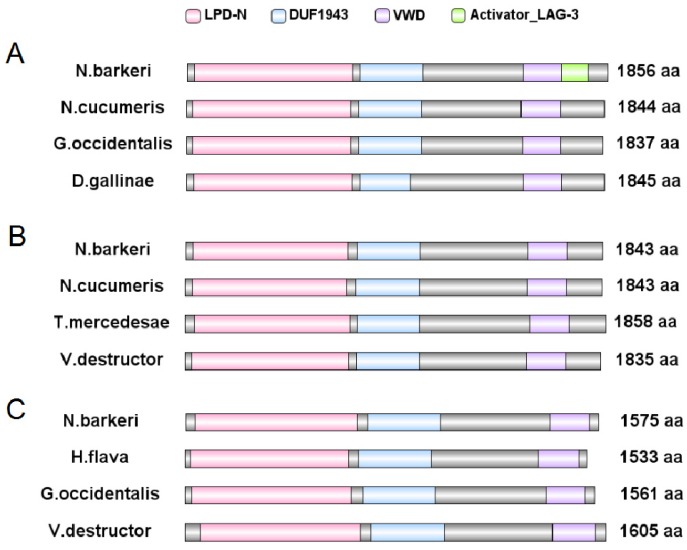
Schematic comparison of the primary protein structures of *N. barkeri* vitellogenin (Vg) with those of other mite or tick species. (**A**) Comparison of NbVg1 gene domains against those of other mite Vg genes. (**B**) Comparison of NbVg2 gene domains against those of other mite or tick Vg genes. (**C**) Comparison of NbVg3 gene domains against those of other mite or tick Vg genes. VWD, Von Willebrand factor D domain; DUF, unknown functional domain; LPD_N, Lipoprotein N-terminal domain

The *N. barkeri* Vg genes include NbVg1, NbVg2, and NbVg3 (GenBank Accession Numbers are ASB34115.1, ASB34116.1, and ASB34117.1, respectively). The length of the open reading frame (ORF) for NbVg1 is 5571 bp, encoding 1856 amino acids (Figure 5A); for NbVg2, the length of the ORF is 5532 bp, encoding 1843 amino acids (Figure 5B); and for NbVg3, the length of the ORF is 4728 bp, encoding 1575 amino acids (Figure 5C). Like those of other mites, the NbVg1, NbVg2, and NbVg3 sequences of *N. barkeri* all contain three conserved regions, LPD_N, DUF 1943, and VWD (Figure 5). However, NbVg1 also has a small domain, Activator_LAG_3, which is involved in regulating growth and development. NbVg1, NbVg2, and NbVg3 all have signal peptides. The signal peptides for NbVg1 and NbVg2 are cleaved between amino acids 16 and 17, and the signal peptides for NbVg3 are cleaved between amino acids 30 and 31. The molecular weights of NbVg1, NbVg2, and NbVg3 are 212, 211, and 179 kDa, and their pI values are 8.61, 8.98, and 6.88, respectively (Table 2).

**Figure 6:**
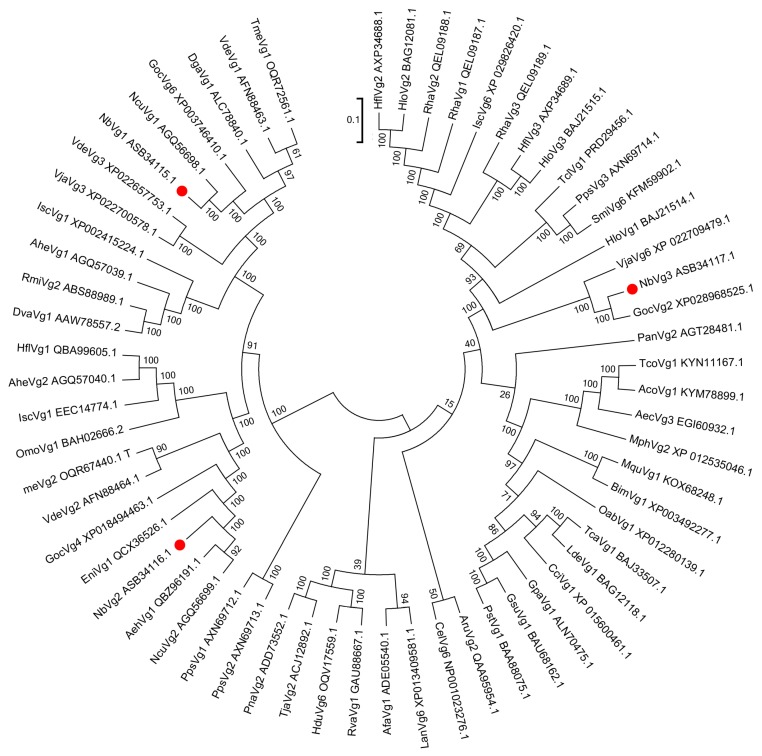
Phylogenetic tree of *N. barkeri* Vgs constructed using the neighbor-joining method in MEGA software (version: 7.0). Each branch shows bootstrap values from 1000 replications. Tables S1–S3 list the Vgs sequences used in this analysis.

A phylogenetic tree was constructed using MEGA 7.0 software (Tempe, AZ, USA) [26] and the neighbor-joining method was used to analyze the relationships between NbVg1, NbVg2, NbVg3, and the Vgs of other Acarine species with those of *N. barkeri*. Figure 6 shows that the sequences of NbVg1, NbVg2, and NbVg3 for *N. barkeri* are clustered with those of *Neoseiulus cucumeris*, *Galendromus occidentalis*, *Varroa destructor*, *Dermanyssus gallinae*, *Hemicordulia flava*, *Planococcus citri*, and *Tetranychus urticae* (Tables S1–S3). The amino acid sequence of NbVg1 has a relatively high homology with those of the *G. occidentalis* and *N. cucumeris* Vgs. The amino acid sequence of NbVg2 is highly homologous to those of the Vgs of *N. cucumeris* and *V. destructor*. The amino acid sequence of NbVg3 is highly homologous to that of the Vgs in *G. occidentalis*. In addition, Arachnida and Insecta Vgs form separate branches.

#### 3.3.2. VgR Bioinformatics Analysis

**Figure 7:**
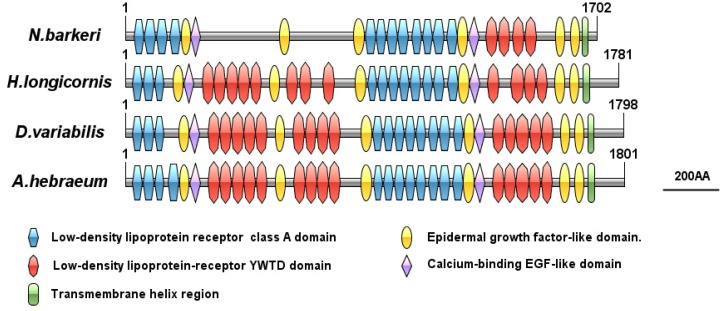
Schematic comparison of primary protein structures for the *N.barkeri* VgR with those of other mite species.

The full length of the VgR (GenBank Accession Number XP_028968361.1) gene sequence in *N. barkeri* is 5335 bp, and the length of the ORF is 5109 bp, encoding 1702 amino acids. The theoretical molecular weight is 191.14 kDa, while the pI is 5.53 (Table 2), and there are eight putative glycosylation sites (N-X-S or N-X-T). The presumed signal peptide is cleaved at amino acid 19. Predicted conserved domain analysis of the VgR amino acid sequence from *N. barkeri* is shown in Figure 7. The NbVgR gene encodes four classical functional domains: LBDs, theEGF precursor homology domain, the transmembrane domain, and the intracellular domain. NbVgR has two LBDs, containing four and eight class a repeat domains, respectively. The EGF precursor domain is located C-terminal to the ligand binding domain, and includes an EGF-like repeat domain and a YWTD motif (Tyr-Trp-Thr-Asp sequence) used to form a β-propeller domain. The NbVgR has two EGF precursor homology domains, each containing four EGF-like repeat domains. However, unlike the proteins encoded by the corresponding genes for *Haemaphysalis longicornis*, *Dermacentor variabilis,* and *Amblyomma hebraeum*, NbVgR does not contain the YWTD motif in the first LBD. NbVgR and that of *Haemaphysalis longicornis* do not contain O-connected structural regions.

**Figure 8:**
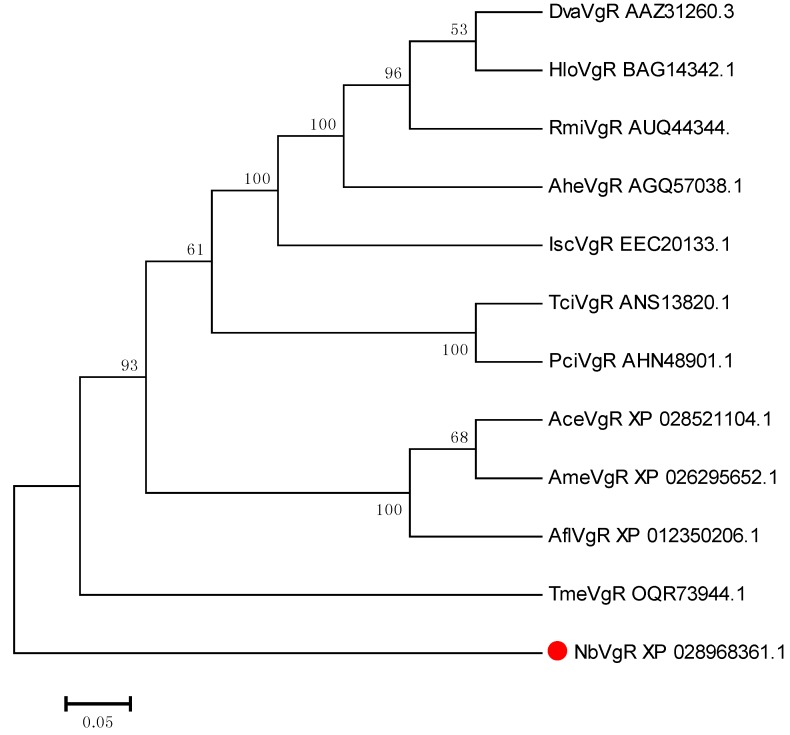
Phylogenetic tree for the VgR in *N. barkeri* constructed using the neighbor-joining method in MEGA software (version: 7.0). Each branch shows bootstrap values from 1000 replications. Table S4 lists the Vgs sequences used in this analysis.

From the phylogenetic trees of NbVgR and the VgR for other species, we can see that arachnids, insects, crustaceans, and vertebrates form independent VgR branches. Among them, NbVgR belongs to Phytoseidae, Tetranychuidae, and Ixodidae, which form three separate groups. This shows that the VgR is highly evolutionarily conserved (Figure 8).

#### 3.3.3. Transcriptional Expression of the NbVgs and NbVgR

**Figure 9:**
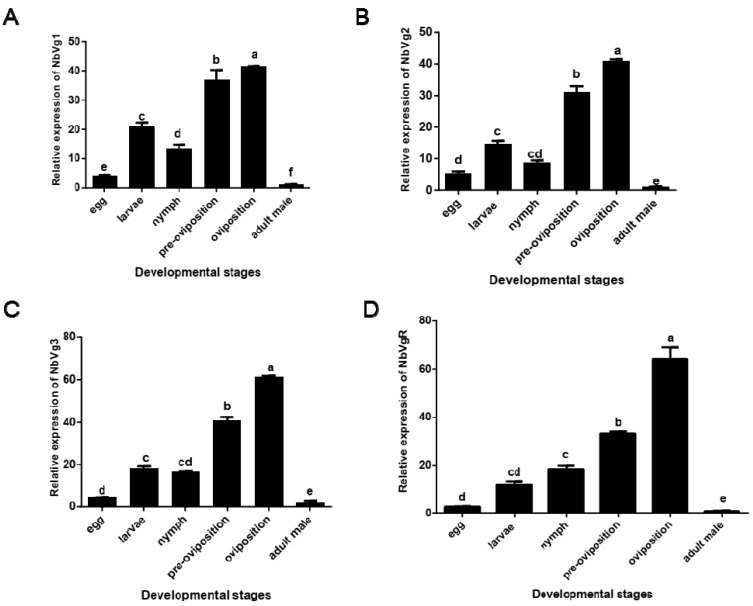
Relative expression of *N. barkeri* Vgs and the VgR in non-diapause mites at different stages as determined by quantitative polymerase chain reaction (qPCR). (**A**) Relative expression of Vg1. (**B**) Relative expression of Vg2. (**C**) Relative expression of Vg3. (**D**) Relative expression of the VgR. Data are presented as the means of three replicates ± standard error (n = 3). Different lowercase letters indicate significant differences (one-way analysis of variance (ANOVA) with Duncan new multiple range method, *p* < 0.05).

Figure 9 shows that there are significant differences in the expression levels of NbVg1, NbVg2, NbVg3, and NbVgR in the eggs, larvae, nymphs, preovipositional female mites (the first day of adult mites), ovipositional female mites (the fourth day of adult mites), and male adult mites. The expression levels for male mites are the lowest, followed by those of the eggs. In the development stage, the expression levels for female adult mites in the preovipositional period are significantly lower than those of female adult mites in the ovipositional period.

**Figure 10:**
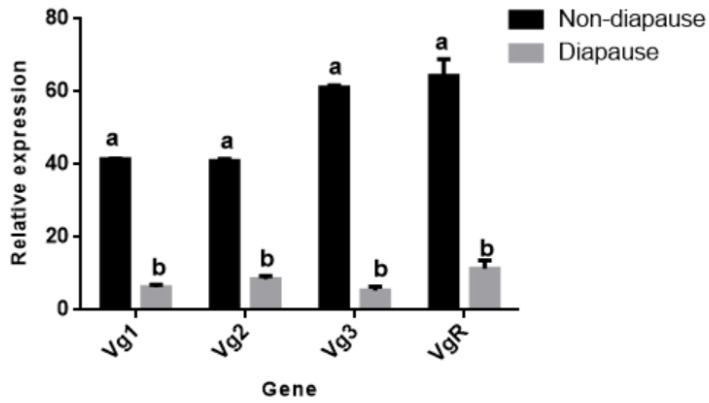
Expressions of *N. barkeri* Vgs and the VgR in non-diapause and diapause periods. Data are presented as the mean ± standard error (n = 3). Different lowercase letters indicate significant differences (one-way ANOVA with Duncan new multiple range method, *p* < 0.05).

Figure 10 shows the expression levels of different genes in the non-diapause female adult *N. barkeri* (the fourth day of adulthood) and the diapause female adult *N. barkeri* (the fifth day of adulthood). The expression levels of NbVg1, NbVg2, NbVg3, and NbVgR are significantly different, and the expression levels for diapause female adult mites are down-regulated.

## 4. Discussion

The main characteristic of diapause in Phytoseius mites is non-oviposition. The abdominal examination of diapause female adult *N. barkeri* mites showed that the ovaries inside the mite are underdeveloped. No yolk granules are formed in the ovaries of diapause female adult mites at any time, and the ovaries of diapause female adult mites remain at first-day development into adulthood (this stage is equivalent to the first day of a non-diapause mated female adult mite, that is, the ovaries have just begun to develop, oocytes and vegetative cells have appeared, but no yolk particles are observed), while the ovaries of non-diapause female adult mites have developed normally by the third day of adulthood (there are obvious yolk granules) and produced eggs. Therefore, this study demonstrates that the ovarian development of diapause female adult mites is halted in stage I. 

Morphology and behavior are also used as criteria for defining reproductive diapause. The external morphological characteristics of diapause female adult *N. barkeri* are a flattened dorsum and abdomen, while in non-diapause females, the abdomen is rounded and enlarged and the ovary has rounded eggs. This feature is similar to other species of Phytoseid mites. For example, the external morphological characteristics of Phytoseius mites, such as *G. occidentalis* and *Amblyseius fallacis*, can be used to identify reproductive diapause [27].

The ORFs of Vg1, Vg2, and Vg3 are 5571, 5532, and 4728 bp, respectively. Bioinformatic analysis showed that the amino acid sequences of NbVg1, NbVg2, and NbVg3 all have characteristic domains of arthropod Vg, such as LPD_N, DUF 1943, and VWD, which comprise three traditionally conserved regions. The N-terminal of NbVg2 contains the lipoprotein domain LPD_N. Studies have shown that LPD_N may be involved in lipid interactions. The VWD protein is the carrier of coagulation factor VIII [28].

The ORF length of the VgR is 5109 bp, encoding 1702 amino acids. The VgR belongs to the LDLR superfamily. As expected, like other members of the LDLR family, the highly conserved domains of NbVgR include the following: (i) the class A cysteine enrichment repeat (ligand binding repeat); (ii) the class B cysteine-rich repeat sequence (EGF repeat sequence); (iii) the transmembrane domain; and (iv) the intracellular domain. NbVgR does not contain O-linked sugar domains, similar to H1VgR in *H. longicornis* and PcVgR in *P. citri*. O-linked sugar domains represent oocyte-specific transcripts, and the existence of this domain is not common in the LDLR family [29].

We measured the transcriptional expression levels of Vg1, Vg2, Vg3, and the VgR in different developmental stages (eggs, young mites, nymphs, preovipositional female adults, ovipositional female adults, diapause female adults, and male adults). For these genes, the expression levels for adult mites are significantly higher than those for the other developmental stages, which is consistent with the trend of other Acarine species, such as *P. citri*, *H. longicornis*, and *A. hebraeum* [30].

The expression levels of Vg1, Vg2, Vg3, and the VgR for mated female adult mites in diapause are significantly lower at similar developmental time points for non-diapause mated female adult mites, because the Vg content in ovaries is affected by Vg biosynthesis and VgR uptake to female germ cells. The decrease of VgR content leads to the inability of oocytes to absorb enough Vg, resulting in reproductive diapause and an inability to lay eggs. NbVgR has a similar expression pattern to the expression of VgR for other insects. Its expression is very low at the beginning of the pre-ovarian stage and significantly increases before the full yolk stage. For example, the highest expression level of *B. dorsalis* (GenBank Accession Number JX469118) VgR appears on the seventh day, and the expression level in the early stage is significantly lower than that on the seventh day [31].

The expression trend of Vg and VgR in diapause and non-diapause *N. barkeri* to some extent reflects its physiological function, that is, Vg and VgR genes are involved in yolk deposition into ovarian oocytes, providing Vg as a nutrient reserve material for the maturation of oocytes [32,33]. Vg and VgR genes are responsible for oviposition in *N. barkeri* and other oocytes. This process is crucial. The results of real-time fluorescent quantitative PCR showed that the relative expression of the Vg gene is significantly higher in sexually mature and ovipositing females than in immature females. Liu et al. reported that Vg gene mRNA is detected in *Chrysopa pallens* at the age of four days and is most accumulated in *C. pallens* at the age of 10 days, after which its Vg gene mRNA expression begins to decrease [34]. After silencing the Vg gene of *C. pallens* with RNAi technology, the number of eggs laid decreases significantly, and the hatchability of the eggs also decreases significantly. This indicates that the Vg gene plays an important role in the laying and hatching of *C. pallens*. In addition, the transcription levels of Vg and the VgR have also been measured in male adult mites, revealing that low levels of Vg and the VgR are also expressed in some insect males, such as *Apis mellifera* [35]. However, Vg is not expressed in the spider mite (*T. urticae*) [36]. Thus, the role of Vg and the VgR in males is unclear and needs further study.

The VgR plays an important role in the development of eggs and is generally considered to be an effective factor in evaluating female reproductive capacity. At present, the synthesis process for Vg is still controversial. There are two main ways to synthesize Vg: (a) It is synthesized in extraovarian tissues, secreted into the circulatory system, and reaches the oocyte. It then enters the ovary through endocytosis mediated by the VgR and provides nutrients for the development of embryos and larvae [37]. (b) The endogenous synthesis of Vg, where Vg is produced by the oocyte itself with the participation of relevant organelles [38]. There is a certain correlation between the expression of Vg and the number of eggs laid by insects. For example, the overexpression of Vg and its receptors in *Tetranychus cinnabar* may cause increased fecundity [39].

## 5. Conclusions

In summary, histological observation revealed marked differences between the ovaries of non-diapause females and those in reproductive diapause. Specifically, the abdomens of fertile females are enlarged and round, while those of females in reproductive diapause are flat. Moreover, the boundaries of eggs can be clearly seen in the ovaries of non-diapause females, along with yolk particles. Conversely, the ovaries of females in reproductive diapause are not completely developed, thus they lack eggs. Given the fact that Vg and the VgR play important roles in reproduction and oviposition, it is not surprising that the expression of Vg and the VgR is significantly higher in fertile females than those in reproductive diapause. This indicates that, like that of *Drosophila* [40], the reproductive capacity of *N. barkeri* is closely related to Vg and the VgR. Specifically, upregulation of Vg and the VgR in mites leads to an increase in Vg and VgR protein content in the fat body and ovaries, providing more nutrients for oviposition. However, the specific mechanism of this process requires further study.

## Figures and Tables

**Table 1:** Primers used for quantitative real-time reverse transcription polymerase chain reaction (qRT-PCR). Vg, vitellogenin; VgR, vitellogenin receptor.

Primer	Sequence (5′–3′)	Product Size (bp)
NbVg1-qF	CGACATTCCCATCTACGC	155
NbVg1-qR	AACCTTCCTGCTCCTTACC	
NbVg2-qF	CGCCAGGAAGAAGGTATC	187
NbVg2-qR	TGTGAGTGGGGCAAACG	
NbVg3-qF	GCCAGCGACCAACAGAT	177
NbVg3-qR	GCGGAAGCAAGGGTAAT	
NbVgR-qF	CACAAGAGGGCGAAGAGC	117
NbVgR-qR	CCATCGGAGCAGAGTCAAG	
NbActin-qF	TCAGCGATGTCAGTTTGAGG	103
NbActin-qR	CCTCCTCTCGCAATGAGAAC	

**Table 2:** Primary structure analysis of NbVg1, NbVg2, NbVg3, and NbVgR.

Protein	Amino Acids	Molecular Weight (kDa)	Theoretical pI
*Nb*Vg1	1856	212	8.61
*Nb*Vg2	1843	211	8.98
*Nb*Vg3	1575	179	6.88
*Nb*VgR	1702	191	5.53

## Supplementary Materials

The following are available online at https://www.mdpi.com/2075-4450/11/4/203/s1, Figure S1: Collection device of *Neoseiulus barkeri*; Table S1: BLAST alignment of the Vg1 gene of *Neoseiulus barkeri* with other species in the NCBI database; Table S2: BLAST alignment of the Vg2 gene of *Neoseiulus barkeri* with other species in the NCBI database; Table S3: BLAST alignment of the Vg3 gene of *Neoseiulus barkeri* with other species in the NCBI database; Table S4: BLAST alignment of the VgR gene of *Neoseiulus barkeri* with other species in the NCBI database.

## References

[1] Hessein N.A., Parrella M.P. (1991). Predatory mites help control thrips on floriculture crops. Calif. Agric..

[2] Grafton Cardwell E.E., Ouyang Y., Striggow R.A. (1999). Predacious mites for control of citrus thrips, *Scirtothrips citri* (Thysanoptera: Thripidae) in nursery citrus. Biol. Control.

[3] Nomikou M., Janssen A., Schraag R., Sabelis W. (2001). Phytoseiid predators as potential biological control agents for *Bemisia tabaci*. Exp. Appl. Acarol..

[4] Fernando L.C.P., Waidyarathne K.P., Perera K.F.G., Silva P.H.P.R.D. (2010). Evidence for suppressing coconut mite, *Aceria guerreronis* by inundative release of the predatory mite, *Neoseiulus baraki*. Biol. Control.

[5] Endong W., Xuenong X., Shengyong W. (2010). Control effects of *Amblyseius barkeri* on *Frankliniella occidentalis* on the eggplants and their natural enemy *Orius sauteri* in the greenhouse. Plant Prot..

[6] Xia B., Zou Z., Li P., Lin P. (2012). Effect of temperature on development and reproduction of *Neoseiulus barkeri* (acari: Phytoseiidae) fed on aleuroglyphus ovatus. Exp. Appl. Acarol..

[7] Prestwich G.D. (1985). Comprehensive insect physiology, biochemistry, and pharmacology. Int. J. Biochem..

[8] Gotoh T. (1986). Annual Life Cycle of the Two-Spotted Spider Mite, *Tetranychus urticae* KOCH (Acarina: Tetranychidae), on *Ribes rubrum* L. in Sapporo: The Presence of Non-Diapausing Individuals. Appl. Entomol. Zool..

[9] Wysoki M. (1974). Studies on diapause and the resistance to low temperatures of a predacious mite, *Phytoseius finimus* (mesostigmata, phytoseiidae). Eniomol. Exp. Appl..

[10] Croft B.A. (1971). Comparative Studies on Four Strains of *Typhlodromus occidentalis* (Acarina: Phytoseiidae). V. Photoperiodic Induction of Diapause. Ann. Entomol. Soc. Am..

[11] Lu K., Shu Y.H., Zhou J.L., Zhang X.Y., Zhang X.Y., Chen M.X., Yao Q., Zhou Q., Zhang W.Q. (2015). Molecular characterization and RNA interference analysis of vitellogenin receptor from *Nilaparvata lugens*. J. Insect Physiol..

[12] Tufail M., Takeda M. (2008). Molecular characteristics of insect vitellogenins. J. Insect Physiol..

[13] Sappington T.W., Raikhel A.S. (1998). Molecular characteristics of insect vitellogenins and vitellogenin receptors. Insect Biochem. Mol. Biol..

[14] Huo Y., Liu W.W., Zhang F.J., Chen X.Y., Li L., Liu Q.F., Zhou Y.J., Wei T.Y., Fang R.X., Wang X.F. (2014). Transovarial transmission of a plantvirus is mediated by vitellogen of its insect vector. PLoS Pathog..

[15] Ge L.Q., Wu J.C., Zhao K.F., Chen Y., Yang G.Q. (2010). Induction of *Nlvg* and suppression of *Nljhe* gene expression in *Nilaparvata lugens* (Stål) (hemiptera: Delphacidae) adult females and males exposed to two insecticides. Pestic. Biochem. Physiol..

[16] Schip F.D.V.H., Samallo J., Broos J., Ophuis J., Mojet M., Gruber M., Geert A.B. (1987). Nucleotide sequence of a chicken vitellogenin gene and derived amino acid sequence of the encoded yolk precursor protein. J. Mol. Biol..

[17] Wahli W., Dawid I.B., Ryffel G.U., Wyler T., Jaggi R.B., Weber R. (1979). Vitellogenin in *Xenopus laevis* is encoded in a small family of genes. Cell.

[18] Kawakami Y., Goto S.G., Ito K., Numata H. (2009). Suppression of ovarian development and vitellogenin gene expression in the adult diapause of the two-spotted spider mite *Tetranychus urticae*. J. Insect Physiol..

[19] Blumenthal T., Squire M., Kirtland S., Cane J., Donegan M., Spieth J., Sharrock W. (1984). Cloning of a yolk protein gene family from *Caenorhabditis elegans*. J. Mol. Biol..

[20] Sappington T.W., Raikhel A.S. (2005). Insect vitellogenin/yolk protein receptors. Reprod. Biol. Invertebr..

[21] Okabayashi K., Shoji H., Nakamura T., Hashimoto O., Asashima M., Sugino H. (1996). cDNA cloning and expression of the *Xenopus laevis* vitellogenin receptor. Biochem. Biophys. Res. Commun..

[22] Raikhel A.S., Dhadialla T.S. (1992). Accumulation of yolk proteins in insect oocytes. Annu. Rev. Entonzol..

[23] Zhong R., Ding T.B., Niu J.Z., Xia W.K., Liao C.Y., Dou W., Wang J.J. (2015). Molecular characterization of vitellogenin and its receptor genes from citrus red mite, *Panonychus citri* (McGregor). Int. J. Mol. Sci..

[24] Ding L., Chen F., Luo R., Pan Q., Wang C., Yu S., Cong L., Liu H., Li H., Ran C. (2018). Gene cloning and difference analysis of vitellogenin in *Neoseiulus barkeri* (Hughes). Bull. Entomol. Res..

[25] Tang Q.Y., Zhang C.X. (2013). Data Processing System (DPS) software with experimental design, statistical analysis and data mining developed for use in entomological research. Insect Sci..

[26] Kumar S., Stecher G., Tamura K. (2016). Mega7: Molecular evolutionary genetics analysis version 7.0 for bigger datasets. Mol. Biol. Evol..

[27] Veerman A. (1992). Diapause in phytoseiid mites: A review. Exp. Appl. Acarol..

[28] Shikina S., Chen C.J., Chung Y.J., Shao Z.F., Liou J.Y., Tseng H.P., Lee Y.H., Chang C.F. (2013). Yolk formation in a stony coral *Euphyllia ancora* (Cnidaria, Anthozoa): Insight into the evolution of vitellogenesis in nonbilaterian animals. Endocrinology.

[29] Alexander D.S., Kaufman W.R. (2014). Molecular characterization of the vitellogenin receptor from the tick, *Amblyomma hebraeum* (Acari: Ixodidae). Insect Biochem. Mol. Biol..

[30] Chippendale G.M., Yin C.M. (1976). Endocrine interactions controlling the larval diapause of the southwestern corn borer, *Diatraea grandiosella*. J. Insect Physiol..

[31] Lin C., Yang W.J., Jiang X.Z., Niu J.Z., Shen G.M., Ran C., Wang J.J. (2015). The Essential Role of Vitellogenin Receptor in Ovary Development and Vitellogenin Uptake in *Bactrocera dorsalis* (Hendel). Int. J. Mol. Sci..

[32] Taylor D., Chinzei Y., Miura K., Ando K. (1991). Vitellogenin synthesis, processing and hormonal regulation in the tick, *Ornithodoros parkeri* (Acari: Argasidae). Insect Biochem..

[33] Qian C., Fu W.W., Wei G.Q., Wang L., Liu Q.N., Dai L.S., Sun Y., Zhu B.J., Liu C.L. (2015). Identification and expression analysis of vitellogenin receptor from the Wild Silkworm, *Bombyx mandarina*. Arch. Insect Biochem. Physiol..

[34] Liu C., Mao J., Zeng F. (2015). *Chrysopa septempunctata* (Neuroptera: Chrysopidae) Vitellogenin Functions through Effects on Egg Production and Hatching. J. Econ. Entomol..

[35] Guidugli-Lazzarini K.R., Nascimento A.M.D., Tanaka E.D., Piulachs M.D., Hartfelder K., Márcia G.B., Paulino L.Z.S. (2008). Expression analysis of putative vitellogenin and lipophorin receptors in honey bee (*Apis mellifera* L.) queens and workers. J. Insect Physiol..

[36] Kawakami Y., Numata H. (2013). Effects of a Pyrethroid on Ovarian Development in Diapause Females of the Two Spotted Spider Mite. J. Acarol. Soc. Jpn..

[37] Okumura T., Yamano K., Sakiyama K. (2007). Vitellogenin gene expression and hemolymph vitellogenin during vitellogenesis, final maturation, and oviposition in female kuruma prawn, *Marsupenaeus japonicus*. Comp. Biochem. Physiol. Part A Mol. Integr. Physiol..

[38] Avarre J.C., Michelis R., Tietz A., Lubzens E. (2003). Relationship between vitellogenin and vitellin in a marine shrimp (*Penaeus semisulcatus*) and molecular characterization of vitellogenin complementary DNAs. Biol. Reprod..

[39] Liu X., Shen G., Xu H., He L. (2016). The fenpropathrin resistant *Tetranychus cinnabarinus* showed increased fecundity with high content of vitellogenin and vitellogenin receptor. Pestic. Biochem. Physiol..

[40] Schonbaum C.P., Perrino J.J., Mahowald A.P. (2000). Regulation of the vitellogenin receptor during *Drosophila melanogaster* oogenesis. Mol. Biol. Cell.

