# Supplementary material for: Molecular Characterization of Neoseiulus barkeri Vitellogenin Genes and Vitellogenin Receptor during Reproductive Diapause
Source: Insects. 2020 Mar 26;11(4):203. doi: 10.3390/insects11040203 (PMC7240613; doi:10.3390/insects11040203)
Supplement: Supplementary file 1 [file insects-11-00203-s001.pdf]

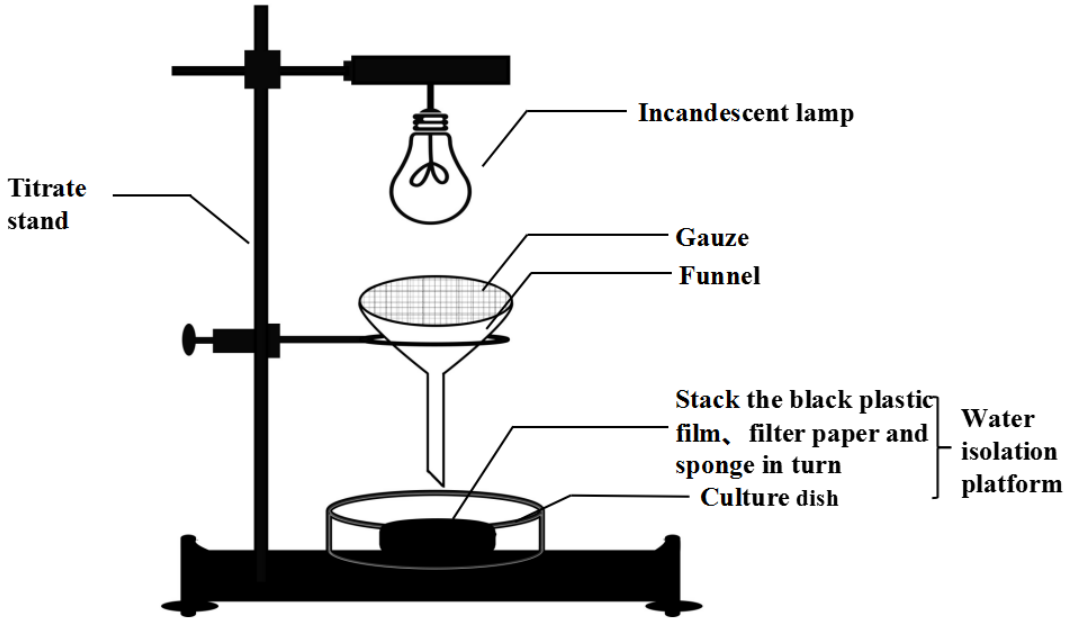

Figure S1. Collection device of *Neoseiulus barkeri.*

Table S1. BLAST alignment of the Vg1 gene of *Neoseiulus barkeri* with other species in the NCBI database.

Species	Protein Size (aa)	Identity(%)	GenBank Accession Number
Neoseiulus cucumeris	1844	77.50	AGQ56698.1
Galendromus occidentalis	1837	70.74	XP_003746410.1
Dermanyssus gallinae	1845	59.49	ALC78840.1
Varroa destructor	1850	57.28	AFN88463.1
Tropilaelaps mercedesae	1840	58.36	OQR72561.1
Ixodes scapularis	1936	31.56	EEC18889.1
Amblyomma hebraeum	1904	31.54	AGQ57039.1
Dermacentor variabilis	1843	30.37	AAW78557.2
Rhipicephalus microplus	602	28.21	ABS88989.1
Varroa jacobsoni	1812	58.75	XP_022700578.1
Varroa destructor	1812	58.69	XP_022657753.1
Ixodes scapularis	1936	31.56	EEC18889.1
Galendromus occidentalis	1850	23.19	XP_018494463.1
Varroa destructor	1835	22.87	AFN88464.1
Ornithodoros moubata	1834	21.64	BAH02666.2
Amblyomma hebraeum	1926	23.14	AGQ57040.1
Pardosa pseudoannulata	1792	22.20	AXN69712.1
Pardosa pseudoannulata	1734	21.55	AXN69713.1
Lingula anatina	1816	22.49	XP_013409581.1
Haemaphysalis flava	1965	25.96	QBA99605.1
Glaucias subpunctatus	1885	23.80	BAU68162.1
Lethocerus deyrollei	1895	22.95	BAG12118.1
Melipona quadrifasciata	1995	22.16	KOX68248.1
Bombus impatiens	1772	21.82	XP_003492277.1
Geocoris pallidipennis	1848	22.43	ALN70475.1
Cephus cinctus	1895	23.11	XP_015600461.1

Table S2. BLAST alignment of the Vg2 gene of *Neoseiulus barkeri* with other species in the NCBI database.

**Species**	**Protein Size (aa)**	**Identity(%)**	**GenBank Accession Number**
Amblyseius eharai	1852	86.04	QBZ96191.1
Panonychus citri	1851	20.21	AHN48900.1
Tetranychus urticae	1749	20.70	XP_015793836.1
Tetranychus truncatus	1755	20.88	AYV88983.1
Tetranychus cinnabarinus	1757	21.87	AMO02571.1
Ornithodoros moubata	1834	31.75	BAH02666.2
Haemaphysalis flava	1965	30.66	QBA99605.1
Varroa destructor	1835	55.95	AFN88464.1
Neoseiulus cucumeris	1843	88.03	AGQ56699.1
Pardosa pseudoannulata	1734	22.35	AXN69713.1
Tigriopus japonicus	1800	21.04	ACJ12892.1
Pseudodiaptomus annandalei	1772	21.55	AGT28481.1
Caenorhabditis elegans	1603	21.41	NP_508589.1
Trichonephila clavipes	1605	20.28	PRD29456.1
Euseius nicholsi	1845	79.00	QCX36526.1
Galendromus occidentalis	1850	76.32	XP_018494463.1
Tropilaelaps mercedesae	1858	58.79	OQR67440.1
Amblyomma hebraeum	1926	31.47	AGQ57040.1
Ixodes scapularis	1644	32.49	EEC14774.1
Varroa jacobsoni	1812	23.71	XP_022700578.1
Asterias rubens	1930	21.17	QAA95954.1
Pseudodiaptomus annandalei	1772	21.55	AGT28481.1

Table S3. BLAST alignment of the Vg3 gene of *Neoseiulus barkeri* with other species in the NCBI database.

Species	Protein Size (aa)	Identity(%)	GenBank Accession Number
Haemaphysalis flava	1533	24.41	AXP34689.1
Haemaphysalis longicornis	1545	24.49	BAG12081.1
Galendromus occidentalis	1561	60.66	XP_028968525.1
Varroa jacobsoni	1575	41.40	XP_022709479.1
Ixodes scapularis	1539	25.40	XP_029826420.1
Rhipicephalus haemaphysaloides	1548	25.53	QEL09188.1
Haemaphysalis longicornis	1694	23.28	BAJ21514.1
Pardosa pseudoannulata	1672	22.13	AXN69714.1
Haemaphysalis longicornis	1462	24.65	BAJ21515.1
Trichonephila clavipes	1605	23.20	PRD29456.1
Stegodyphus mimosarum	1564	21.34	KFM59902.1
Rhipicephalus haemaphysaloides	1270	26.87	QEL09187.1
Caenorhabditis elegans	1651	21.08	NP_001023276.1
Azumapecten farreri	2296	26.64	ADE05540.1
Paracyclopina nana	1813	18.96	ADD73552.1

Table 4. BLAST alignment of the VgR gene of *Neoseiulus barkeri* with other species in the NCBI database.

Species	Protein Size (aa)	Identity(%)	GenBank Accession Number
Dermacentor variabilis	1798	28.27	AAZ31260.3
Rhipicephalus microplus	1799	28.34	AUQ44344.1
Apis mellifera	1754	26.83	XP_026295652.1
Eufriesea mexicana	1737	27.64	OAD52717.1
Araneus ventricosus	1721	26.71	GBM68820.1
Limulus polyphemus	1666	32.86	XP_022244855.1
Ixodes scapularis	1200	32.25	EEC20133.1
Amblyomma hebraeum	1801	32.43	AGQ57038.1
Apis cerana	1526	26.09	XP_028521104.1
Tropilaelaps mercedesae	1613	31.08	OQR73944.1
Haemaphysalis longicornis	1781	31.55	BAG14342.1
Tetranychus cinnabarinus	1852	31.72	AMO02573.1
Panonychus citri	1891	32.17	AHN48901.1
